# Reactive Oxygen Species-Induced Impairment of Endothelium-Dependent Relaxations in Rat Aortic Rings: Protection by Methanolic Extracts of *Phoebe grandis*

**DOI:** 10.3390/molecules16042990

**Published:** 2011-04-06

**Authors:** Lau Yeh-Siang, Gopal Subramaniam, A. Hamid A. Hadi, Dharmani Murugan, Mohd Rais Mustafa

**Affiliations:** 1 Centre of Natural Products and Drug Discovery (CENAR), Department of Pharmacology, Faculty of Medicine, University of Malaya, 50603 Kuala Lumpur, Malaysia; E-Mails: lauyehsiang@yahoo.com (L.Y.-S.); kadagam@yahoo.com (G.S.); dharmani79@um.edu.my (D.M.); 2 Centre of Natural Products and Drug Discovery (CENAR), Department of Chemistry, Faculty of Science, University of Malaya, 50603 Kuala Lumpur, Malaysia; E-Mail: ahamid@um.edu.my

**Keywords:** *Phoebe grandis*, β-NADH, isolated rat aorta, endothelial dysfunction, superoxide anions

## Abstract

Generation of reactive oxygen species plays a pivotal role in the development of cardiovascular diseases. The present study describes the effects of the methanolic extract of *Phoebe grandis* (MPG) stem bark on reactive oxygen species-induced endothelial dysfunction *in vitro*. Endothelium-dependent (acetylcholine, ACh) and -independent relaxation (sodium nitroprusside, SNP) was investigated from isolated rat aorta of Sprague-Dawley (SD) in the presence of the β-NADH (enzymatic superoxide inducer) and MPG extract. Superoxide anion production in aortic vessels was measured by lucigen chemiluminesence. Thirty minutes incubation of the rat aorta *in vitro* with β-NADH increased superoxide radical production and significantly inhibited ACh-induced relaxations. Pretreatment with MPG (0.5, 5 and 50 μg/mL) restored the ACh-induced relaxations (R_max_: 92.29% ± 2.93, 91.02% ± 4.54 and 88.31 ± 2.36, respectively) in the presence of β-NADH. MPG was ineffective in reversing the impaired ACh-induced relaxations caused by pyrogallol, a non-enzymatic superoxide generator. Superoxide dismutase (a superoxide scavenger), however, reversed the impaired ACh relaxations induced by both β-NADH and pyrogallol. MPG also markedly inhibited the β-NADH-induced generation of the superoxide radicals. Furthermore, MPG scavenging peroxyl radicals generated by tBuOOH (10^−4^ M).These results indicate that MPG may improve the endothelium dependent relaxations to ACh through its scavenging activity as well as by inhibiting the NADH/NADPH oxidase induced generation of superoxide anions.

## 1. Introduction

The vascular endothelium plays a pivotal role in regulating normal vascular tone and maintaining uninterrupted blood flow in the vessels [1]. Under normal conditions, the endothelium regulates vascular homeostasis by releasing a variety of factors that act locally in the blood vessel wall and lumen, such as nitric oxide (NO), prostacyclin and endothelin. Nitric oxide (NO) released from the endothelium in response to various vasoactive factors such as ACh plays a key role in the maintenance of smooth muscle relaxation. Vascular endothelial dysfunction may be defined as impairment in endothelium dependent vasodilatation and alteration in the normal properties of endothelium [2]. The vascular dysfunction results in reduced activation of endothelial nitric oxide synthase (eNOS), and reduced generation and bioavailability of nitric oxide (NO) [3]. Among the factors contributing to the endothelial dysfunction is the overproduction of reactive oxygen species such as superoxide anions (O_2_^-^) which binds and inactivates NO released from the endothelium [4].

*Phoebe grandis (Nees) Merr* is a local Malaysian timber tree of some 20 m is height with yellowish brown flowers [5]. Mukhtar *et al.* [6] recently demonstrated that the stem bark of *P. grandis* contains several known isoquinoline alkaloids: (-)-8,9-dihydrolinearisine, boldine, norboldine, lauformine, scortechine A and scortechine B and a novel oxoproaporphine; (-)-grandine A, Two of the aporphine alkaloids, boldine and norboldine have been demonstrated to have several pharmacological actions including anti-inflammatory, anti-cancer, antidiabetic and potent antioxidant activities [6,7,8]. Plants rich in antioxidants are much sought out for their therapeutic potential, particularly in the prevention of cardiovascular diseases such as atherosclerosis, heart failure and hypertension. Several studies with these pytochemicals, including green tea, showed improved endothelial function by inhibiting O_2_^-^ production or scavenging the O_2_^-^ anions [9,10,11,12]. In the present study, we investigated the endothelial effect of the methanolic extract of *Phoebe grandis* (MPG) on the NADH/NADPH oxidase induced oxidative stress in the isolated rat thoracic rat aorta.

## 2. Results and Discussion

### 2.1. tBuOOH-induced intracellular oxidative stress

**Figure 1:**
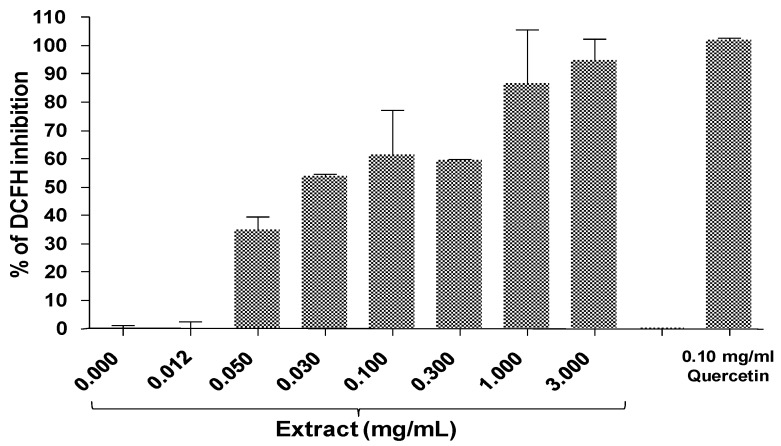
Effect of various concentration *P.grandis* (stem bark) methanolic extract on oxidative stress induced by 10^−4^ M tBuOOH. Quercetin (0.1 mg/mL) was used as positive control.

In the present study, the MPG extract protected the cells against the oxidative stress effect of superoxide anions radical generated by tBuOOH (10^−4^ M). At the highest concentration, MPG (3 mg/mL) inhibited almost completely the oxidative stress induced by tBuOOH (Figure 1).

### 2.2. Effect of MPG extract on vascular relaxations

**Figure 2:**
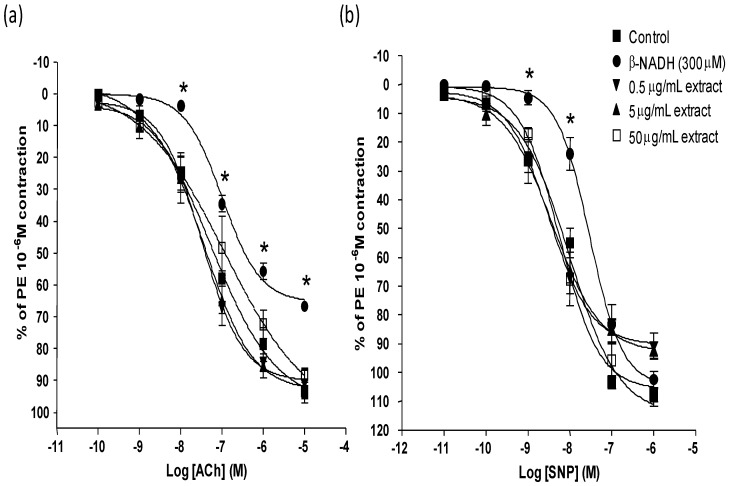
Effect of 0.5 μg/mL, 5 μg/mL and 50 μg/mL *P.grandis* (stem bark) methanolic extract on (a) ACh and (b) SNP relaxation in rat aortic rings induced oxidative stress by β-NADH. Results are mean ± S.E.M (n = 5–6). Significant difference from control is indicated by * (p < 0.05).

Concentration dependent vasorelaxation was observed with both ACh and SNP. Maximal relaxation induced by ACh at 10^−5^ M and SNP at 10^−6^ M was 93.36% ± 3.82 and 105.09 % ± 3.87, respectively (Figure 2a and 2b).

Presence of β-NADH attenuated ACh induced relaxation (R_max_ 66.64% ± 1.25) and reduced the responses to SNP (10^−9^–10^−7^ M) without marked effects on maximal relaxation (R_max_ 98.67% ± 3.94) (Figure 2a and 2b). Presence of β-NADH slightly decreased the sensitivity of ACh and SNP (Table 1).

Pre-incubation with MPG extract alone or the vehicle (0.1% DMSO), did not affect the vascular responses to ACh (data not shown). MPG extract also did not affect the resting tension of the aortic rings or the KCl-induced contraction. In the presence of β-NADH, pre-incubation with different concentrations of MPG extracts (0.5, 5 and 50 μg/mL) significantly improved the ACh induced relaxations (R_max_: 92.29% ± 2.93, 91.02% ± 4.54 and 88.31 ± 2.36, respectively) (Figure 2a). In the presence of β-NADH, pre-incubation of 50, 5 or 0.5 μg/mL MPG extract did not significantly affect the SNP maximal relaxation compared to the control (Figure 2b). The different concentrations of the extract only slightly reversed the decreased sensitivity of ACh and SNP which was seen in the presence of β-NADH (Table 1).

**Figure 3:**
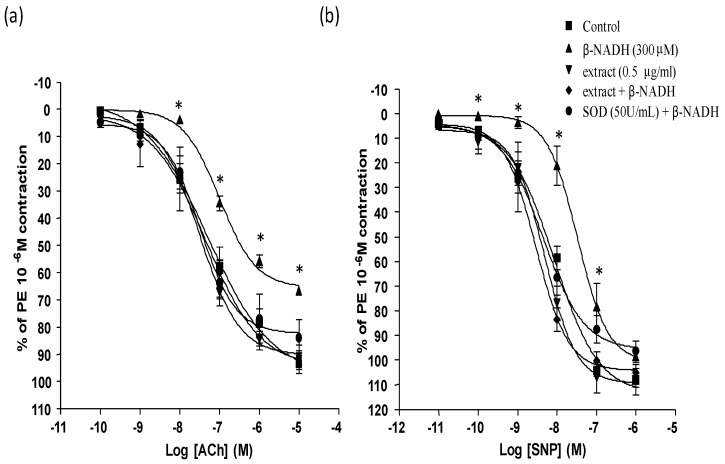
Effect of SOD, a superoxide scavenger and 0.5 μg/mL *P.grandis* (stem bark) methanolic extract in the presence of β-NADH on (a) ACh and(b) SNP- induced relaxation in rat aortic rings. Results are mean ± S.E.M (n = 5–6). Significant difference from control is indicated by * (p < 0.05).

**Figure 4:**
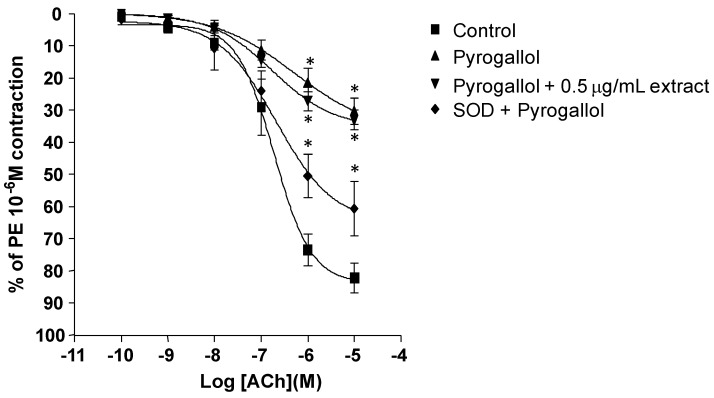
Effect of SOD, a superoxide scavenger and 0.5 μg/mL *P.grandis* (stem bark) methanolic extract in the presence of pyrogallol (non-enzymatic superoxide inducer) on Ach-induced relaxation in rat aortic rings. Results are mean ± S.E.M (n = 5–6). Significant difference from control is indicated by * (p < 0.05).

In the presence of SOD and β-NADH, ACh-induced relaxation was significantly improved compared with the group with β-NADH alone. The improvement in ACh relaxation was similar to those observed with 0.5 μg/mL extract (Figure 3a). ACh- induced relaxation was impaired in the presence of 10 μM pyrogallol with maximal relaxation at 10^−6^ M ACh 30.33% compared to control 93.36%. The pEC_50_ of ACh-induced relaxation in the presence of pyrogallol was not significantly altered compared to the control (−6.30 ± 0.68 *vs* −7.18 ± 0.13, respectively). Preincubation of 0.5 μg/mL extract did not alter the impaired relaxation caused with pyrogallol (pEC_50_, −6.76 ± 0.23). However, pre-incubation of the aorta with SOD, markedly reduced the inhibitory effects of pyrogallol on the ACh-induced relaxation of the aorta (pEC_50_, −6.67 ± 0.34) (Figure 4).

### 2.3. Detection of superoxide anion

**Figure 5:**
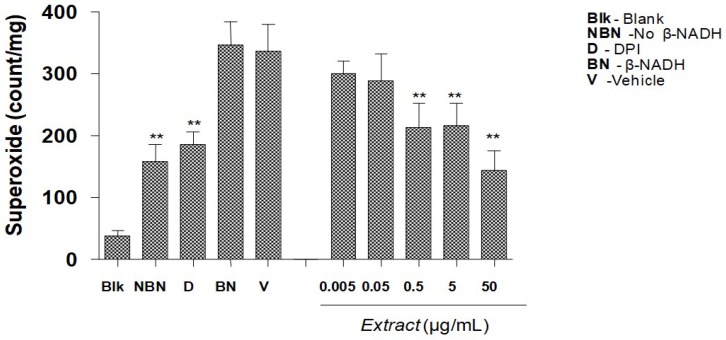
Effect of various concentration of *P.grandis* (stem bark) methanolic extract onsuperoxide production induced by β-NADH in rat aortic rings detected by chemilunescenceassay. Results are mean ± S.E.M (n = 5–7). Significant difference from vehicle control is indicated by ** (p < 0.01).

Measurement of superoxide anion by lucigenin chemiluminesence assay demonstrated that MPG dose-dependently decreased superoxide anion production induced by β-NADH (Figure 5). Without the presence of β-NADH, the superoxide radical production by rat aortic ring was 100 ± 50 counts/mg. In the presence of β-NADH, the superoxide radical production increased to 350 ± 50 counts/mg. DPI, an inhibitor of NADPH oxidase, significantly decreased the superoxide radical production induced by β-NADH (200 ± 25 counts/mg). The vehicle used to dissolve the extract did not have any effect on the superoxide production induced by β-NADH. The MPG extract dose-dependently reduced the superoxide anion stimulated by β-NADH with a significant decrease observed from the concentration 0.5 μg/mL to 50 μg/mL.

### 2.4. Discussion

The results from the present study shows that the stem bark of MPG exhibited: (1) a marked cellular antioxidant activity at the concentration range of 1–3 mg/mL and (2) improved endothelium dependent relaxation by inhibiting NADH/NADPH oxidase stimulated superoxide production. In the cell-based assay, the intracellular antioxidant effects of the MPG extract protected the cells against the reactive oxygen species, *i.e.* peroxyl radicals, induced by tBuOOH. One of the active components of MPG extract is boldine, an aporphine alkaloid, and known to possess potent peroxyl radical scavenging and poor superoxide scavenging functions [7]. The cell-based assay reaffirms that the MPG extract posses free radical scavenging activity. Improved the endothelium-dependent relaxations to ACh in response to oxidative stress induced by β-NADH and decreased tissue superoxide levels, suggests that MPG is able to scavenge peroxyl radicals as well as reduce the generation of superoxide radicals produced by intracellular NADH/NADPH oxidase.

Accumulating evidence indicates that the generation of reactive oxygen species (ROS) is closely associated with the development of many cardiovascular diseases [13]. β-NADH has been commonly used to stimulate NADH/NADPH oxidase activity *in vitro*. Previous studies have indicated increased superoxide generation in vascular vessel preparations in response to application of exogenous NADH [14,15,16]. In the present study, preincubation of the tissues with β-NADH markedly reduced the endothelium dependent relaxations to ACh in the isolated rat aorta and partly the endothelium independent relaxation to SNP. Stimulation of M_3_-muscarinic receptor by ACh releases nitric oxide from the endothelium which then diffuses into adjacent smooth muscle cells and leads to soluble guanylate cyclase (sGC) activation, cyclic GMP elevation and ultimately to vascular smooth muscle relaxation [17]. SNP breaks down spontaneously to yield nitric oxide, thereby causing endothelium-independent vasodilatation by the same effector mechanism as nitric oxide released from endothelium (*i.e.* activation of sGC) [17]. Increased production of superoxide anions, leads to reduce released of nitric oxide and/or inactivation of nitric oxide released from the endothelium, and ultimately attenuating the ACh-induced relaxations and partly SNP-induced relaxation.

Pretreatment with MPG significantly prevented β-NADH-induced attenuation of ACh relaxation in the rat aorta, suggesting the extract may increase the bioavailability of NO from the scavenging effects of the oxygen radicals. Furthermore, treatment with the extract in β-NADH treated aortas partly improved the relaxant responses and the sensitivity to endothelium-independent nitric oxide donor, SNP. This is indirect evidence that the beneficial effect of MPG on ACh-induced relaxation resides mainly improving the upstream endothelial nitric oxide bioavailability, since the extract only partly attenuated the downstream nitric oxide signal transduction pathway.

Several studies have repeatedly shown that treatment with antioxidants improved endothelium-dependent relaxations in animal models of oxidative stress such as in spontaneously hypertensive rats [12,18]. Many medicinal plants have also been found to cause endothelium-dependent relaxation in vascular tissues. Many are related to the balance between NO and superoxide anions [19]. In the present study, the MPG extract protected the cells against the oxidative stress effect of reactive oxygen radicals generated by tBuOOH. Boldine, an aporphine alkaloid is found in high concentrations in *Phoebe grandis*, and is known to possess potent antioxidative and free radical scavenging functions [20]. Thus, antioxidant actions of MPG may have increased the bioavailability of endothelium-derived nitric oxide, subsequently increasing the ACh-dependent relaxations. Both MPG and superoxide anions scavenger (sodium dismutase), improved ACh-induced relaxation in β-NADH pretreated tissues with an essentially similar magnitude. This indicates that MPG may be scavenging the β-NADH-induced superoxide productions in the rat aorta.

However, in the presence of pyrogallol, MPG failed to restore the NO-dependent relaxations. Pyrogallol, a prooxidant auto-oxidizes in tissue bath medium to generate extracellular superoxide anion, while β-NADH stimulates release of intracellular superoxide anions from NADH/NADPH oxidase activity [15,21]. This is suggesting that the protective effect of the extract does not involve scavenging of superoxide anions but somewhat affects the intracellular NADH/NADPH oxidase activity in endothelial cells or in the vascular smooth muscle cells. These hypotheses is further supported in which measurement of superoxide anion by lucigenin chemiluminesence assay demonstrated that MPG dose-dependently decreased superoxide anion production induced by β-NADH. This suggests that MPG may inhibit NADH/NADPH oxidase stimulated superoxide production in a similar manner to apocynin [22].

## 3. Experimental

### 3.1. Preparation of MPG extract

Bark of *Phoebe grandis (Nees)* Merr. (Lauraceae) were collected at Sik, Kedah (1994) by G. Perromat (Institut de Chimie des Substances Naturelles, CNRS, Gif sur Yvette, France). Identification was made by Dr K. M. Kochummen (Forest Research Institute of Malaysia, Kepong, Malaysia). Voucher specimens (KL 4318) are deposited at the Laboratoire de Phanerogamie. Museum National d’Historie Naturelle in Paris, at the Herbarium of Department of Chemistry, University of Malaya, Kuala Lumpur, Malaysia and at the Herbarium of the Forest Research Institute, Kepong, Malaysia. A total of 11.5 g of crude extract was obtained from the bark (1 kg). Crude product underwent column chromatography on silica gel with CH_2_Cl_2_ containing increasing amount of methanol and subsequent purification by preparative thin layer chromatography [5].

### 3.2. Cell based intracellular anti-oxidant assay

The intracellular antioxidant activity of the MPG extract was evaluated against the formation of intracellular reactive oxygen species (ROS) in HepG2 cells after treatment with t-BuOOH (tert butyl hydroperoxide), a compound used to induce oxidative stress. The cells were seeded in 96 wells plate at 3 × 10^4^ cells/well (Black plate with transparent bottom) and incubated for 24 h at 37 °C in 5% CO_2_. The next day, various concentrations of MPG extract was added into the wells and incubated again for another 1 h. The MPG extracts were dissolved in DMSO and the final concentration of DMSO (0.2%) used were not toxic to the cells or affect the assay. Next, cells were washed with phosphate buffered saline (PBS) and followed with the incubation with 10 µM dichlorofluorecesin (DCF) for 60 min. Then, cells were washed and incubated with 10^−4^ M tBuOOH for 60 min to induce oxidative stress. The plate was read at 485/535 nm. Results were expressed as a percentage inhibition of control [23].

### 3.3. Preparation of aortic rings

Twelve weeks old male Sprague-Dawley (SD) rats were obtained from the University of Malaya animal house and housed in well ventilated room at ambient temperature. They were provided with normal rat chow and tap water *ad libitum*. All experiments were reviewed and approved by the University of Malaya Animal Care and Ethics Committee. The rats were killed by cervical dislocation and aorta from the thoracic region was excised and cleared from any adherent fat and connective tissue with extra care to avoid any damage to the endothelium. The thoracic aorta was cut into small rings (3–5 mm in width) and suspended in a 5 mL organ bath containing Krebs physiological salt solution (pH 7.4) of the following composition (mM): NaCl 118, KCl 4.7, CaCl_2_·2H_2_O 2.5, KH_2_PO_4_ 1.2, MgSO_4_.7H_2_O 1.2, glucose 11.7, NaHCO_3_ 25.0, and EDTA 0.026. The tissue-bath solution was aerated continuously with 95% oxygen and 5% carbon dioxide at 37 °C. Isometric tension (g) was measured using a force displacement transducer connected to a Mac Lab recording system (ADI Instruments, Australia). The aortic rings were allowed to equilibrate for 20 min under resting tension of 1 g before any initiation of experimental protocols. Each experiment was conducted with a minimum of 5–8 number of rats.

### 3.4. Pharmacological studies

After equilibration, the rings were repeatedly stimulated with KCl solution (high K^+^, 80 mM) for 4 min at 10 min intervals until two consecutive equal contractions were reached. Following washout of high K^+^ responses, vascular relaxation study was performed by doing cumulative concentration–response curves to the endothelium-dependent and -independent relaxant agonists, acetylcholine (ACh, 10^−10^ to 10^−5^ M) and sodium nitroprusside (SNP, 10^−11^ to 10^−6^ M), respectively. To test the relaxation responses to ACh and SNP, the aortic rings were pre-contracted with phenylepherine, PE (1 μM). To investigate the involvement of O_2_^-^ on vascular relaxation, 300 μM β-NADH (induces O_2_^-^ through NADH/NADPH oxidase) was preincubated for 30 min prior to conducting concentration curve to ACh and SNP. To investigate the scavenging ability of MPG extract, various concentration of the extract (0.5, 5 or 50 μg/mL) was incubated with β-NADH for 30 min prior to ACh and SNP concentration response study. In other experiments, pyrogallol (10 μM) was incubated with the aorta to generate the superoxides independent of NADH/NADPH oxidase. SOD (50 UI/mL), a superoxide scavenger was used as a positive control. Pre-incubation with β-NADH, SOD and pyrogallol did not affect the resting tension of the aortic tissue. Indomethacin (10 μM) was added in all experiments to exclude the influence of prostaglandin.

### 3.5. Measurement of superoxide anions

Lucigenin-enhanced chemiluminescence assay is performed as described previously by Chan *et al.* [24] with some modification. The aortic rings were pre-incubated at 37 °C in Krebs-Hepes buffer containing diethylythiocarbamic acid (DETCA, 10 mM) and β-NADH (0.3 mM) and either vehicle (20% Tween 80), MPG extract (0.005 µg/mL–50 µg/mL) or diphenylene iodonium (DPI, NADPH oxidase inhibitor) for 45 minutes. The rings were then transferred to a 96-well plate in luminometer (Plate CHAMELEONTM, Hidex, Finland). The background photon was measured previously for 20 min in the presence of 5 μM of lucigenin and Krebs-Hepes buffer. The output of chemiluminescence is then measured for 20 min. All of the samples are dried in a 65 °C oven for 48 h. The results are expressed as counts per milligram dry weight tissue (*i.e.,* count/ mg).

### 3.6. Calculations and statistical analysis

The concentrations indicated in the text or in the figures represent the final tissue-bath concentrations of respective drugs. The responses were recorded as mean ± standard error of the mean (S.E.M.) and ‘n’ indicates number of rats used for each set of data. Statistical evaluation of the data for pharmacological studies and chemiluminescense assay was performed by unpaired Student’s *t*-test when comparing means of two groups and one-way analysis of variance (ANOVA) and Dunnett post hoc test for more than two group comparisons. A value of *p* < 0.05 was considered statistically significant.

### 3.7. Chemicals and drugs

Dichlorofluorecesin, *tert*-butylhydroperoxide (tBuOOH), ACh, SNP, β-NADH, SOD, pyrogallol, DECTA and DPI were purchased from Sigma Aldrich Chemicals. HepG2 was purchased from ATCC (American Type Culture Collection). Chemicals used for Krebs solution preparation were purchased from BDH.

## 4. Conclusions

In summary, results from the present study showed that MPG extract may have improved the magnitude of endothelial dependent and independent relaxations through its antioxidant activity (peroxyl radical scavenging) as well as inhibiting NADH/NADPH induced superoxide anion productions in endothelial cells or vascular smooth muscle cells.

## Figures and Tables

**Table 1:** pEC_50_ and R_max_ value for ACh- and SNP- induced relaxation in rat aortic rings pre-treated with various concentrations of MPG, β-NADH and SOD. Results are mean ± S.E.M (n = 4–6). Significant difference from control is indicated by * (p < 0.05).

	ACh		SNP	
	pEC_50_(M)	R_max_ (%)	pEC_50_(M)	R_max_ (%)
Control	-7.29 ± 0.13	93.36 ± 3.83	-8.01 ± 0.11	105.09 ± 3.87
0.5 μg/mL extract	-7.49 ± 0.19	92.96 ± 2.93	-8.36 ± 0.28	95.33 ± 1.83
5 μg/mL extract	-7.42 ± 0.16	91.02 ± 4.54	-8.40 ± 0.19	93.63 ± 3.19
50 μg/mL extract	-6.94 ± 0.34	88.31 ± 2.36	-8.23 ± 0.11	107.02 ± 4.69
β-NADH (300μM)	-6.99 ± 0.16	66.64 ± 1.25 *	-7.52 ± 0.28	98.67 ± 3.94
β-NADH ± 0.5 μg/mL extract	-7.34 ± 0.27	91.30 ± 2.49	-8.53 ± 0.13	105.21 ± 3.70
β-NADH ± SOD (50 IU/mL)	-7.48 ± 0.18	84.00 ± 6.72	-8.35 ± 0.13	96.33 ± 4.02

## References

[1] Balakumar P., Chakkarwar V.A., Krishan P., Singh M. (2009). Vascular endothelial dysfunction: A tug of war in diabetic nephropathy?. Biomed. Pharmacother..

[2] Stehouwer C.D. (2004). Endothelial dysfunction in diabetes nephropathy: State of the art and potential significance for non diabetic renal disease. Nephrol. Dial. Transplant..

[3] Calles-Escandon J., Cipolla M. (2001). Diabetes and Endothelial dysfunction: A Clinical Perspective. Endocrine Rev..

[4] Berry C., Brosnan M.J., Fennell J., Hamilton C.A., Dominiczak A.F. (2001). Oxidative stress and vascular damage in hypertension. Curr. Opin. Nephrol. Hypertens..

[5] Mukhtar M.R., Martin M.T., Domansky M., Rais M., Hadi A.H., Awang K. (1997). Phoebegrandines A and B, proaporphine-tryptamine dimers, from Phoebe grandis. Phytochemistry.

[6] Mukhtar M.R., Aziz A.N., Thomas N.F., Hadi A.H., Litaudon M., Awang K. (2009). Grandine A, a new proaporphine alkaloid from the bark of Phoebe grandis. Molecules.

[7] O’Brien P., Carrasco-Pozo C., Speisky H. (2006). Boldine and its antioxidant or health promoting properties. Chem. Biol. Interact..

[8] Zhao Q., Zhao Y., Wang K. (2006). Antinociceptive and free radical scavenging activities of alkaloids isolated from Lindera angustifolia. J. Ethnopharm..

[9] Ajay M., Achike F.I., Mustafa A.M., Mustafa M.R. (2006). Effect of quercetin on altered vascular reactivity in aortas from streptozotocin-induced diabetic rats. Diabetes Res. Clin. Pract..

[10] Nakagawa T., Yokozawa T. (2002). Direct scavenging of nitric oxide and superoxide by green tea. Food Chem. Toxicol..

[11] Romero M., Jimenez R., Sanchez M., Lopez-Sepulveda R., Zarzeulo M.J., O’Valle F., Zarzuelo A., Pérez-Vizcaíno F., Duarte J. (2009). Quercetin inhibits vascular superoxide production induced by endothelin-1: Role of NADPH oxidase, uncoupled eNOS and PKC. Arteriosclerosis.

[12] Vera R., Sanchez M., Galista M., Villar I.C., Jimenez R., Zarzuelo A., Pérez-Vizcaíno F., Duarte J. (2007). Chronic administration of genistein improves endothelial dysfunction in spontaneously hypertensive rats: involvement of eNOS, caveolin and calmodulin expression and NADPH oxidase activity. Clin. Sci..

[13] Cai H., Harrison D.G. (2000). Endothelial Dysfunction in Cardiovascular Diseases: The Role of Oxidant Stress. Circ. Res..

[14] Brandes R.P., Barton M., Philippens K.M., Schweitzer G., Mugge A. (1997). Endothelial-derived superoxide anions in pig coronary arteries: evidence from lucigen chemiluminescence and histochemical techniques. J. Physiol. (Lond)..

[15] Didion S.P., Faraci F.M. (2002). Effects of NADH and NADPH on superoxide levels and cerebral vascular tone. Am. J. Physiol. Heart Circ. Physiol..

[16] Lund D.D., Faraci M.M., Miller F.R., Heistad D.D. (2000). Gene transfer of endothelial nitric oxide synthase improves relaxation of carotid arteries from diabetic rabbits. Circulation.

[17] Murad F. (1986). Cyclic guanosine monophosphate as a mediator of vasodilatation. J. Clin. Invest..

[18] Machha A., Mustafa M.R. (2005). Chronic treatment with flavonoids prevents endothelial dysfunction in spontaneously hypertensive rat aorta. J. Cardiovasc. Pharmacol..

[19] Achike F.I., Kwan C.Y. (2003). Nitric oxide, human diseases and herbal products that affect the nitric oxide signalling pathway. Clin. Exp. Pharmacol. Physiol..

[20] Schmeda-Hirschmann G., Rodriquez T.A., Theoduloz C., Astudillo S.L., Feresin G.E., Tapia A. (2003). Free-radical scavengers and antioxidants from Peumus boldus Mol. ("Boldo"). Free Rad. Res..

[21] Marklund S., Marklund G. (1974). Involvement of superoxide anion radical in the autooxidation of pyrogallol and a convenient assay for superoxide dismutase. Eur. J. Biochem..

[22] Stolk J., Hiltermann T.J., Dijkman J.H., Verhoeven A.J. (1994). Characteristics of the inhibition of NADPH oxidase activation in neutrophils by apocynin, a methoxy-substituted catechol. Am. J. Resp. Cell Mol. Biol..

[23] Kim Y.A., Kong C.S., Um Y.R., Lee J.I., Nam T.J., Seo Y. (2008). Antioxidant efficacy of extracts from variety of seaweeds in a cellular system. Ocean Sci. J..

[24] Chan E.C., Drummond G.R., Woodman O.L. (2003). 3’, 4’-dihydroxyflavonol enhances nitric oxide bioavailability and improves vascular function after ischemia and reperfusion injury in the rat. J. Cardiovasc. Pharmacol..

